# Two Faces of the Right Ventricle in Fabry Cardiomyopathy: Septal-Coupled and Atrial-Coupled Strain Patterns and Their Differential Associations with Enzyme Replacement Therapy

**DOI:** 10.3390/biom16081196

**Published:** 2026-08-17

**Authors:** Kuo-Tzu Sung, Dau-Ming Niu, Shu-Fen Hsu, Ming-En Liu, Po-Lin Lin, Yau-Huei Lai, Hsiang-Wei Yang, Yung-Hsiu Lu, Cheng-Ting Tsai, Ta-Chuan Hung, Po-Sheng Chen, Chung-Lieh Hung

**Affiliations:** 1 Institute of Clinical Medicine, National Yang Ming Chiao Tung University, Taipei 112304, Taiwan; 8905012@gmail.com (K.-T.S.);; 2 Division of Cardiology, MacKay Memorial Hospital, Taipei 104217, Taiwan; 3 Department of Medicine, MacKay Medical University, New Taipei City 252005, Taiwan; 4 Telehealth Center, MacKay Memorial Hospital, Taipei 104217, Taiwan; 5 Department of Pediatrics, Taipei Veterans General Hospital, Taipei 112201, Taiwan; 6 School of Medicine, National Yang Ming Chiao Tung University, Taipei 112304, Taiwan; 7 Institute of Molecular and Genomic Medicine, National Health Research Institutes, Miaoli 350401, Taiwan; 8 School of Nursing, MacKay Medical University, Taipei 252005, Taiwan; 9 Division of Cardiology, Hsinchu MacKay Memorial Hospital, Hsinchu 30071, Taiwan; 10 Division of Cardiology, National Cheng Kung University Hospital, Tainan 704302, Taiwan; 11 Institute of Biomedical Sciences, MacKay Medical University, New Taipei City 252005, Taiwan

**Keywords:** Fabry disease, right ventricular strain, enzyme replacement therapy, atrial remodeling, artificial intelligence, automated strain analysis

## Abstract

Right ventricular global longitudinal strain (RVGLS) and right ventricular free-wall longitudinal strain (RVFWLS) are both used to assess right ventricular involvement, but they include different myocardial components. We examined their cross-sectional correlates and longitudinal changes associated with enzyme replacement therapy (ERT) in 100 patients with genetically confirmed Fabry disease, including 69 with serial echocardiography. RVGLS was independently associated with left ventricular global longitudinal strain (LVGLS; standardized β = 0.466, *p* < 0.001) and interventricular septal thickness (standardized β = 0.257, *p* = 0.027), whereas RVFWLS was independently associated with right atrial reservoir strain (standardized β = −0.542, *p* < 0.001). RVGLS and RVFWLS were not significantly correlated (r = 0.053, *p* = 0.654). In the paired RV strain subgroup, longitudinal RVGLS change differed between untreated and ERT-treated patients (+2.06 ± 3.94% vs. −1.28 ± 3.64%, *p* = 0.017), whereas RVFWLS change did not (*p* = 0.838). In a sensitivity model adjusted for age, sex, genotype, and baseline RVGLS, ERT remained associated with ΔRVGLS (β = −3.28 percentage points, 95% CI −5.25 to −1.32; *p* = 0.001). These findings identify different correlates of septal-inclusive and free-wall RV strain and require prospective validation.

## 1. Introduction

Fabry disease is an X-linked lysosomal storage disorder caused by α-galactosidase A deficiency and progressive glycosphingolipid accumulation [1,2]. Cardiac involvement is a major determinant of morbidity and mortality and commonly includes left ventricular (LV) hypertrophy, impaired myocardial deformation, diastolic dysfunction, arrhythmia, and fibrosis [2,3]. These imaging manifestations are clinically relevant because LV hypertrophy, impaired LV global longitudinal strain (LVGLS), and myocardial fibrosis have been associated with adverse outcomes [4]. In East Asian populations, the intronic GLA variant IVS4+919G>A is associated with a predominantly cardiac phenotype [5]. Although surveillance has traditionally focused on the LV, atrial imaging studies indicate early functional involvement [6,7], while right ventricular imaging studies demonstrate right-sided abnormalities that may not parallel conventional measures of RV function [8,9]. Together, these observations support a multichamber rather than exclusively LV-centered view of Fabry cardiomyopathy.

The right ventricle (RV) provides a useful framework for examining this heterogeneity because its systolic performance reflects septal motion, free-wall deformation, loading conditions, and atrioventricular interaction [10,11]. RV strain abnormalities can be detected in Fabry disease even when conventional RV indices remain preserved, particularly in patients with LV hypertrophy [9,12]. Comparative data also suggest that RV strain patterns in Fabry cardiomyopathy differ from those in sarcomeric hypertrophic cardiomyopathy [13]. However, whether septal-inclusive RV global longitudinal strain (RVGLS) and RV free-wall longitudinal strain (RVFWLS) reflect the same remodeling process remains uncertain. This distinction may also affect longitudinal interpretation: a change in RVGLS may not necessarily indicate a parallel change in RV free-wall or atrial-coupled mechanics. RVGLS incorporates septal and free-wall deformation, whereas RVFWLS isolates free-wall deformation [10,14]. We therefore hypothesized that RVGLS would be associated predominantly with LV-septal remodeling and that RVFWLS would be associated more closely with right atrial function, representing two complementary functional patterns rather than interchangeable measures. We also examined RVGLS/pulmonary artery systolic pressure (PASP) and RVFWLS/PASP as exploratory load-adjusted indices. Because the cardiac effects of disease-specific therapy vary across phenotypes and disease stages [15,16], we further evaluated longitudinal changes according to enzyme replacement therapy (ERT) status. A single artificial intelligence-enabled echocardiographic platform, previously externally validated for automated LV strain measurement, was used for standardized analysis across baseline and follow-up studies [17]. Accordingly, this multicenter study evaluated the cross-sectional correlates of RVGLS and RVFWLS and their longitudinal patterns according to ERT status.

## 2. Materials and Methods

### 2.1. Study Design and Population

This multicenter retrospective observational study was a hypothesis-generating investigation of RV remodeling in Fabry cardiomyopathy. Cross-sectional analyses evaluated the structural and functional correlates of RVGLS and RVFWLS; longitudinal analyses assessed changes in these measures according to ERT status. Consecutive patients with genetically confirmed Fabry disease who underwent transthoracic echocardiography at MacKay Memorial Hospital (Taipei, Tamsui, and Hsinchu branches) or National Cheng Kung University Hospital were enrolled. The cross-sectional cohort included 100 patients; 69 had paired serial studies (35 untreated and 34 ERT-treated). Baseline was defined as the last examination before ERT initiation or, for untreated patients, the earliest examination during observation. In Taiwan, reimbursement for ERT requires prior authorization based on documented clinically significant organ involvement; therefore, patients with earlier-stage or subclinical involvement may remain untreated. The study protocol was approved by the institutional review board (IRB No. 25MMHIS408e) and complied with the Declaration of Helsinki. Only patients carrying a pathogenic or likely pathogenic GLA variant were included; no variant of uncertain significance was included. Five GLA variants were represented: c.640-801G>A (IVS4+919G>A), c.658C>T, c.1066C>T, c.1228A>G, and c.413G>A. Variants were reported using transcript NM_000169.3, and clinical classifications reflected the aggregate germline interpretations in ClinVar (accessed 18 July 2026). In female patients, molecular confirmation of a pathogenic or likely pathogenic GLA variant was the primary diagnostic criterion; α-galactosidase A activity and plasma lyso-Gb3 were considered supportive biochemical evidence when available but were not required for inclusion. Patient-level variants, phenotypes, and ERT status are provided in Supplementary Table S1.

### 2.2. Echocardiographic Acquisition

Comprehensive transthoracic echocardiography was performed at participating centers in accordance with contemporary recommendations from the American Society of Echocardiography and the European Association of Cardiovascular Imaging (ASE/EACVI) [10,18]. Standard acquisition included parasternal long- and short-axis views and apical four-, two-, and three-chamber views. Dedicated RV-focused apical four-chamber views were acquired whenever feasible to optimize assessment of RV morphology and function. The protocol included measurements of cardiac structure and function, including LV geometry, systolic and diastolic function, atrial structure and function, RV size and performance, and estimated PASP. Two-dimensional grayscale cine loops and Doppler datasets were stored digitally for offline analysis. For serial examinations, efforts were made to maintain consistent imaging planes and acquisition protocols across follow-up studies.

### 2.3. AI-Enabled Automated Echocardiographic Quantification and Study Definitions

All digitally stored echocardiographic studies were analyzed offline using a single vendor-independent, deep learning-based automated platform (Us2.v2; Us2.ai, Singapore), previously externally validated for automated LV strain measurement [17]. The same platform was used for all baseline and follow-up studies. Standard chamber dimensions, wall thickness, LV mass, LV ejection fraction, Doppler indices, RV dimensions, RV fractional area change, tricuspid regurgitation velocity, and estimated PASP were obtained from the automated analysis and structured output. Myocardial deformation was derived from two-dimensional grayscale cine loops using automated endocardial border detection and frame-to-frame tracking. All measurements were fully automated; no manual contour correction was performed. Investigators visually reviewed each tracing and accepted it for analysis or rejected it when tracking was inadequate. At baseline, RVGLS was available in all 100 studies and RVFWLS in 73 (73%); the remaining 27% were unavailable for RVFWLS-specific analyses because adequate free-wall visualization or tracking could not be achieved. In the longitudinal cohort, paired RVGLS and RVFWLS measurements at both time points were available for a paired longitudinal analysis. For repeat-analysis reproducibility, the same blinded investigator repeated image selection and automated-tracing acceptance in 20 randomly selected studies per measure; no contours were edited. Mean coefficients of variation were 4.4% for RVGLS and 2.8% for RVFWLS.

LVGLS was calculated from the apical four- and two-chamber views. RVGLS was derived from automated tracking in the apical four-chamber view and incorporated both septal and RV free-wall deformation; RVFWLS was derived after automated isolation of the RV free-wall segments. Right and left atrial reservoir strain represented peak positive atrial deformation during ventricular systole. RVGLS/PASP and RVFWLS/PASP were calculated using absolute strain values divided by echocardiographically estimated PASP. ΔRV strain was defined as RVFWLS minus RVGLS. Supplementary Figure S1 shows the automatically generated, color-coded endocardial tracking contours of the RV free wall and interventricular septum together with the corresponding longitudinal strain curve. RVGLS and RVFWLS were analyzed as separate measures rather than as interchangeable indices. More negative ΔRV strain values indicated greater separation between septal-inclusive RVGLS and free-wall RVFWLS. Left ventricular hypertrophy (LVH) was defined using sex-specific ASE/EACVI criteria as LV mass index >115 g/m^2^ in men and >95 g/m^2^ in women [18]. Longitudinal change was calculated as follow-up minus baseline value.

### 2.4. Statistical Analysis

Continuous variables are presented as mean ± standard deviation or median with interquartile range, as appropriate; categorical variables are presented as counts and percentages. Baseline characteristics were compared between untreated and ERT-treated patients using the independent-samples t test or Mann–Whitney U test for continuous variables and the chi-square test or Fisher exact test for categorical variables. Pearson correlations evaluated associations of RVGLS, RVFWLS, ΔRV strain, RVGLS/PASP, and RVFWLS/PASP with LV remodeling, systolic and diastolic function, atrial function, and RV afterload. Multivariable linear regression models for RVGLS and RVFWLS used identical prespecified covariates: age, sex, LVGLS, interventricular septal thickness (IVSd), E/e′ mean, and right atrial (RA) reservoir strain. Standardized regression coefficients were reported to compare effect sizes. Patients were stratified by IVSd tertiles, and ΔRV strain was compared using one-way analysis of variance. In patients with available LV mass index, RVGLS, RVFWLS, RA strain, and ΔRV strain were compared by LVH status, and correlations were repeated within each LVH subgroup. Longitudinal changes were compared between untreated and ERT-treated patients using independent-samples t tests, with standardized mean differences and 95% confidence intervals. Multivariable models with ΔRVGLS as the outcome adjusted for age, sex, and genotype. A sensitivity model additionally adjusted for baseline RVGLS. A sequential model included baseline RVGLS and concurrent ΔLVGLS, in addition to age, sex, and genotype, to assess whether the ERT-associated RVGLS change was independent of concurrent LV mechanical change. Additional sensitivity analyses adjusted directly for follow-up duration and restricted the cohort to follow-up ≥2 years and to the IVS4+919G>A subgroup. Analyses were performed in Stata version 17 (StataCorp, College Station, TX, USA), and visualizations were generated in Python version 3.12.13. Two-sided *p* < 0.05 was considered statistically significant.

## 3. Results

### 3.1. Study Population and Baseline Characteristics

The baseline cohort included 100 patients (66 untreated and 34 ERT-treated); 69 had serial echocardiography. The IVS4+919G>A variant was present in 91 patients, all with a later-onset cardiac phenotype. The remaining nine patients had classical phenotypes and carried c.658C>T (p.Arg220Ter; *n* = 3), c.1066C>T (p.Arg356Trp; *n* = 3), c.1228A>G (p.Thr410Ala; *n* = 2), or c.413G>A (p.Gly138Glu; *n* = 1). Patient-level sex, GLA variant, clinical classification, phenotype, and ERT status are detailed in Supplementary Table S1. Compared with untreated patients, ERT-treated patients had a higher proportion of men (88% vs. 52%, *p* = 0.001) and more advanced cardiac involvement at baseline. They had greater IVSd (13.87 ± 3.78 vs. 10.56 ± 3.17 mm, *p* < 0.001), greater LV mass and relative wall thickness, less negative LVGLS (−14.37 ± 4.20% vs. −17.12 ± 3.96%, *p* = 0.002), and worse diastolic indices. Right-sided differences included larger RVIDd and RA area and less negative RVFWLS, whereas RVGLS, RA reservoir strain, and PASP did not differ significantly (Table 1). Intra-observer reproducibility was good in randomly selected subsets of 20 studies for each parameter (mean coefficient of variation, 4.4% for RVGLS and 2.8% for RVFWLS).

### 3.2. Correlation Analyses of RV Strain Parameters

**Figure 1:**
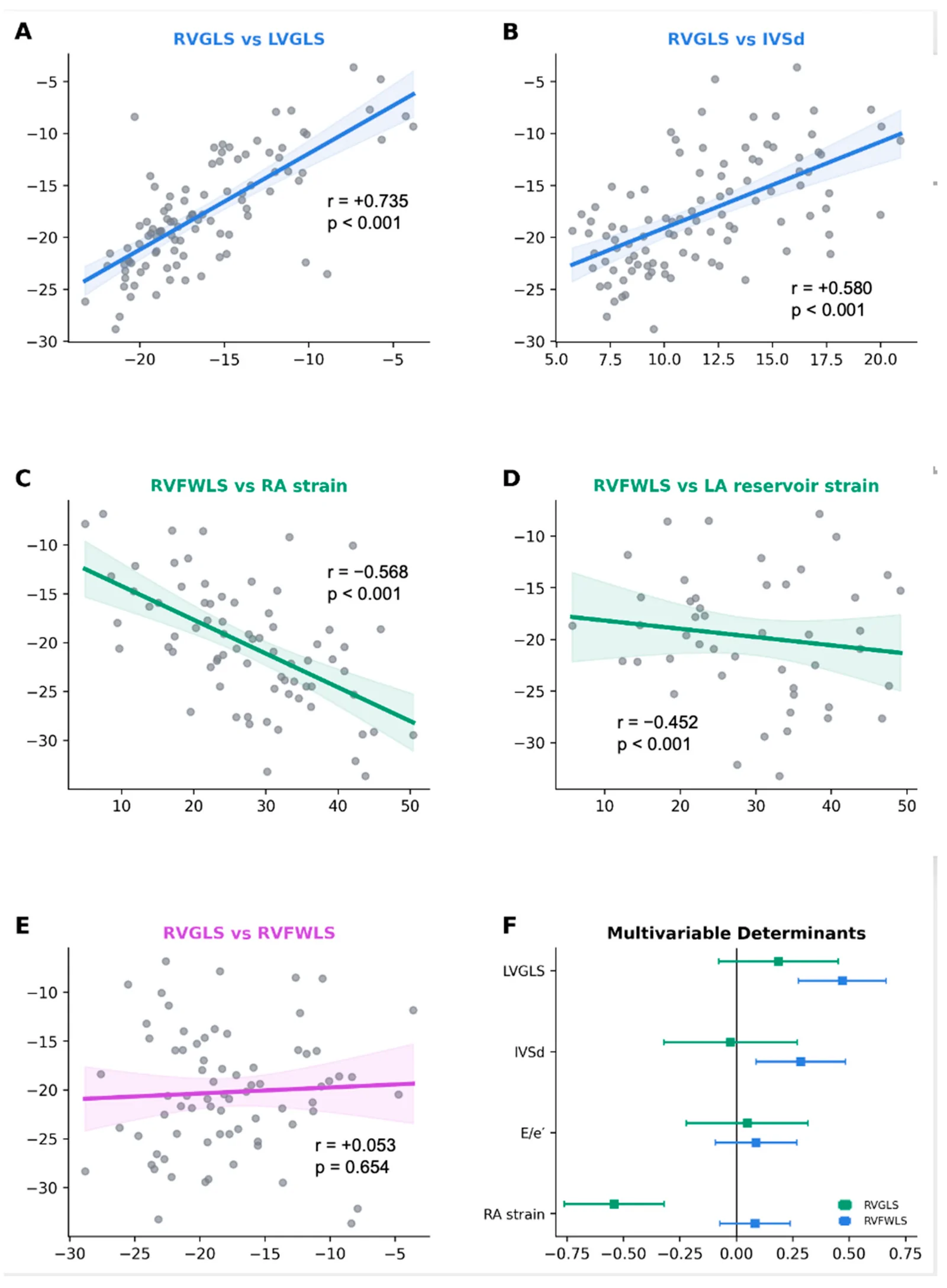
Differential associations of RVGLS and RVFWLS in Fabry cardiomyopathy. (**A**) RVGLS correlated with LVGLS. (**B**) RVGLS correlated with interventricular septal thickness (IVSd). (**C**) RVFWLS correlated inversely with right atrial (RA) reservoir strain. (**D**) RVFWLS correlated inversely with left atrial (LA) reservoir strain. (**E**) RVGLS and RVFWLS were not significantly correlated. (**F**) In multivariable models adjusted for age, sex, IVSd, LVGLS, E/e′ mean, and RA reservoir strain, with identical covariates entered into both models, RVGLS was independently associated with LVGLS and IVSd, whereas RVFWLS was independently associated with RA strain. Shaded bands represent 95% confidence intervals; points represent individual patients. In (**F**), squares indicate standardized regression coefficients and horizontal lines indicate 95% confidence intervals. RVGLS, right ventricular global longitudinal strain; RVFWLS, right ventricular free-wall longitudinal strain; LVGLS, left ventricular global longitudinal strain; IVSd, interventricular septal thickness at end-diastole; RA, right atrium; LA, left atrium.

Correlation analyses are summarized in Table 2 and Figure 1A–E. RVGLS was correlated with LVGLS (r = 0.735, *p* < 0.001), IVSd (r = 0.580, *p* < 0.001), and E/e′ mean (r = 0.431, *p* < 0.001), but not with RA or LA reservoir strain. In contrast, RVFWLS was correlated with RA reservoir strain (r = −0.568, *p* < 0.001) and LA reservoir strain (r = −0.452, *p* < 0.001), but not with LVGLS, IVSd, or E/e′ mean. RVGLS and RVFWLS were not significantly correlated with each other (r = 0.053, *p* = 0.654). These cross-variable findings indicate that the observed associations were selective rather than uniformly shared by both RV strain measures. Complete correlations, including ΔRV strain and the exploratory strain/PASP indices, are provided in Table 2.

### 3.3. Multivariable Determinants of RVGLS and RVFWLS

Multivariable models using identical prespecified covariates are summarized in Table 3 and Figure 1F. LVGLS (standardized β = 0.466, *p* < 0.001) and IVSd (standardized β = 0.257, *p* = 0.027) were independently associated with RVGLS, whereas E/e′ mean and RA reservoir strain were not. The RVGLS model had an adjusted R^2^ of 0.499. RA reservoir strain was the only independent correlate of RVFWLS (standardized β = −0.542, *p* < 0.001); LVGLS, IVSd, and E/e′ mean were not significantly associated with RVFWLS. The RVFWLS model had an adjusted R^2^ of 0.270. Age and sex were not significantly associated with either RV strain measure.

### 3.4. Longitudinal Changes According to ERT Status

**Figure 2:**
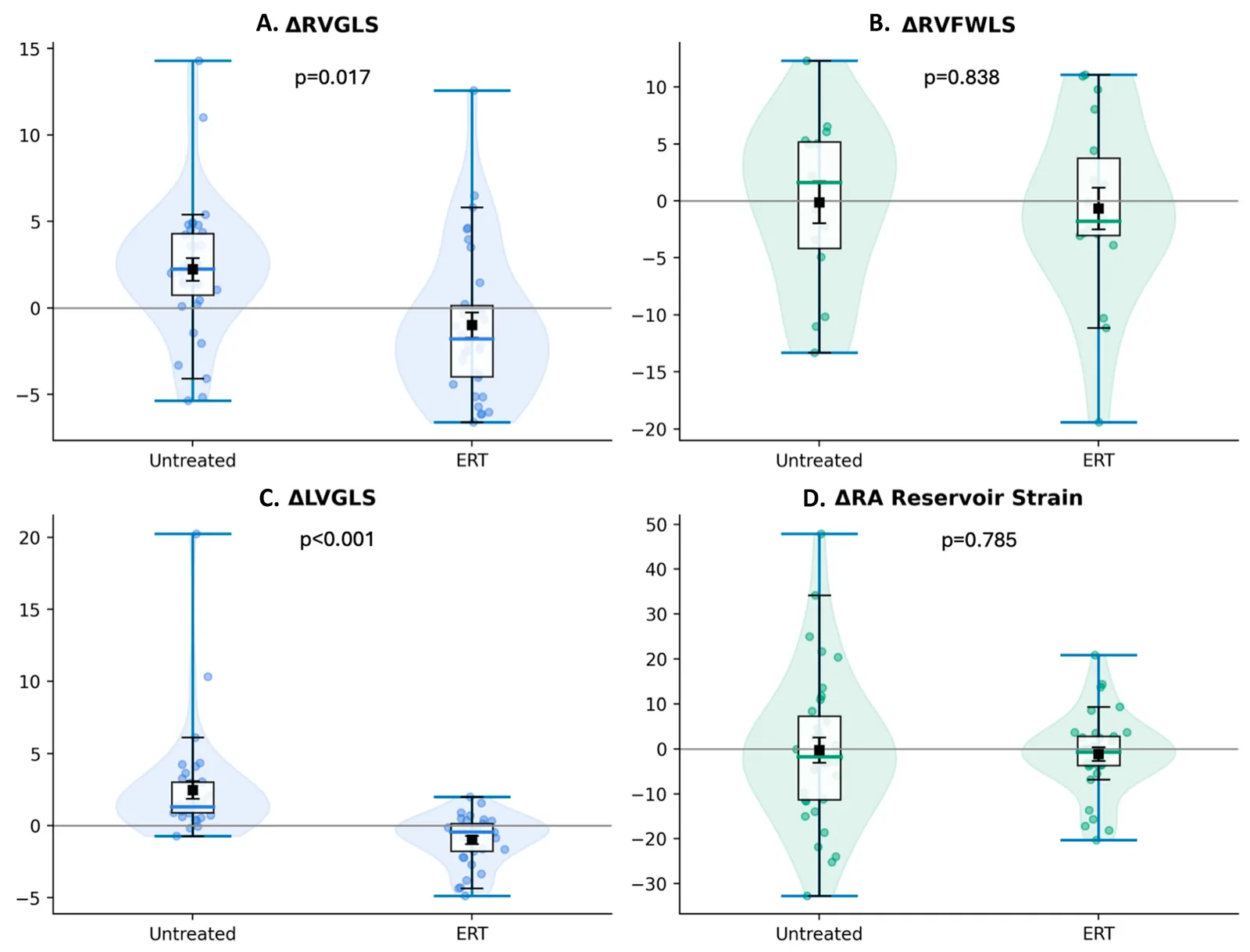
Longitudinal changes in RVGLS, RVFWLS, LVGLS, and RA strain according to ERT status. (**A**) Longitudinal RVGLS change differed between untreated and ERT-treated patients. (**B**) RVFWLS change did not differ significantly between groups. (**C**) LVGLS change differed between groups. (**D**) RA strain change did not differ significantly between groups. Negative Δ values indicate more negative myocardial strain at follow-up. Violin plots show distributions, box plots show medians and interquartile ranges, dots represent patients, and black squares show mean ± standard error. ERT, enzyme replacement therapy; RVGLS, right ventricular global longitudinal strain; RVFWLS, right ventricular free-wall longitudinal strain; LVGLS, left ventricular global longitudinal strain; RA, right atrial; Δ, follow-up minus baseline.

Longitudinal changes are summarized in Table 4 and Figure 2. In the 33 patients with paired RVGLS and RVFWLS measurements, RVGLS change differed between untreated and ERT-treated patients (+2.06 ± 3.94% vs. −1.28 ± 3.64%, *p* = 0.017; standardized mean difference, −0.91 [95% CI −1.67 to −0.15]), whereas RVFWLS change did not (−0.11 ± 7.37% vs. −0.67 ± 7.97%, *p* = 0.838). In the full longitudinal cohort, LVGLS change also differed between groups (+2.49 ± 3.71% vs. −0.56 ± 3.04%, *p* < 0.001). No significant between-group differences were observed for RA reservoir strain (*p* = 0.785), E/e′ mean (*p* = 0.856), PASP (*p* = 0.262), or RVGLS/PASP (*p* = 0.263).

### 3.5. Association of Septal Thickness, LVH Status, and Longitudinal RVGLS Change

**Figure 3:**
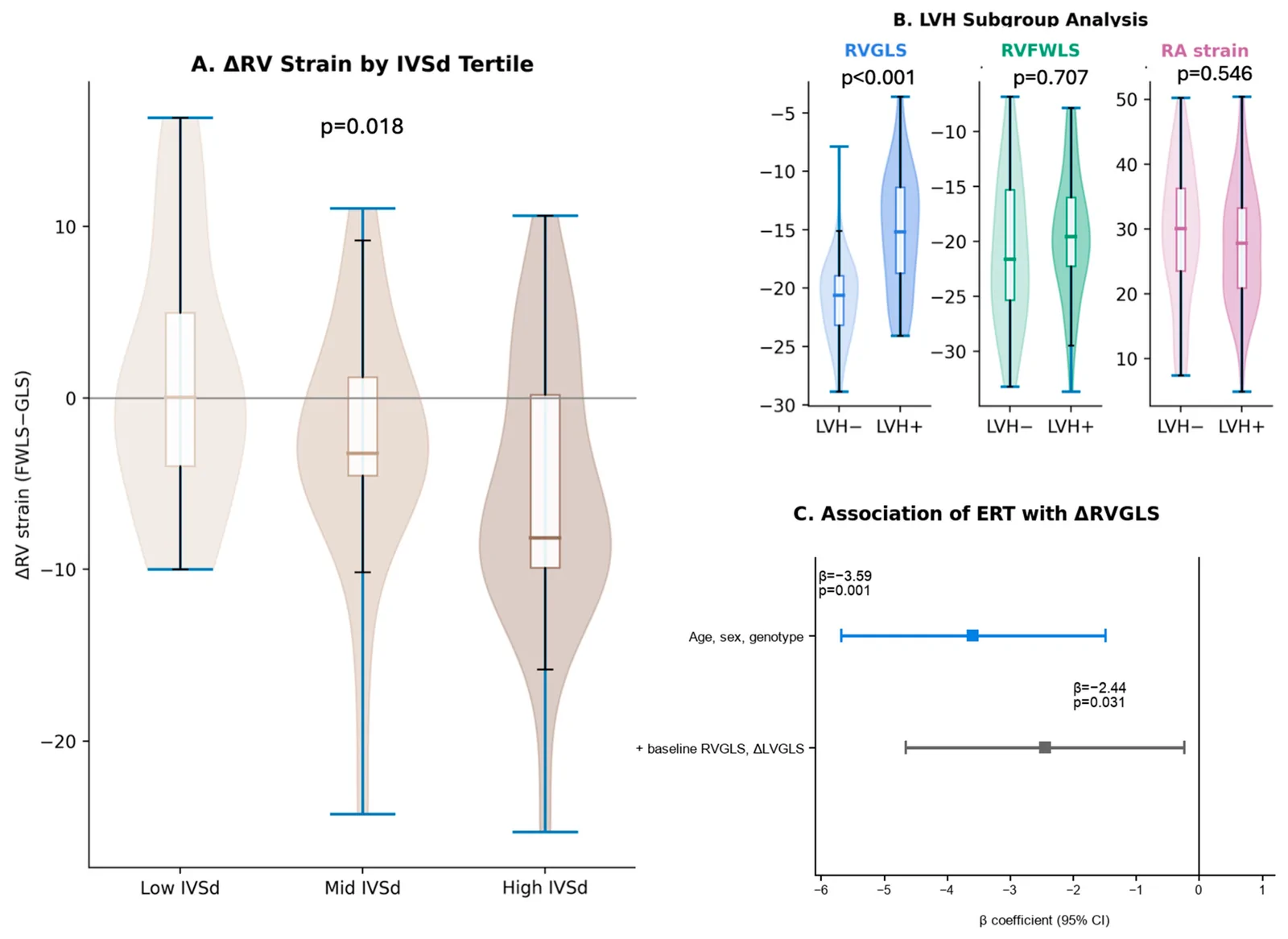
Septal hypertrophy and the difference between RVGLS and RVFWLS. (**A**) ΔRV strain (RVFWLS−RVGLS) differed across IVSd tertiles (≤9.3, 9.3−13.0, and >13.0 mm; ANOVA *p* = 0.018). (**B**) RVGLS was more impaired in LVH-positive patients, whereas RVFWLS and RA strain did not differ significantly by LVH status. (**C**) Association between ERT and ΔRVGLS in the primary model adjusted for age, sex, and genotype and in the sequential model additionally adjusted for baseline RVGLS and concurrent ΔLVGLS. Squares indicate regression coefficients and horizontal lines indicate 95% confidence intervals. ΔRV strain, RVFWLS−RVGLS; RVGLS, right ventricular global longitudinal strain; RVFWLS, right ventricular free-wall longitudinal strain; RA, right atrial; IVSd, interventricular septal thickness at end-diastole; LVH, left ventricular hypertrophy; ERT, enzyme replacement therapy; ΔLVGLS, longitudinal change in left ventricular global longitudinal strain; CI, confidence interval.

ΔRV strain differed across IVSd tertiles (*p* = 0.018; Figure 3A). Among patients with available LV mass index, RVGLS was less negative in those with LVH than in those without LVH (−15.72 ± 5.66% vs. −21.14 ± 3.58%, *p* = 0.0002), whereas RVFWLS (*p* = 0.707) and RA reservoir strain (*p* = 0.546) did not differ by LVH status (Figure 3B). Associations between RVGLS and LVGLS and between RVFWLS and RA strain remained significant within both LVH strata. In the model adjusted for age, sex, and genotype, ERT was associated with ΔRVGLS (β = −3.59 percentage points, 95% CI −5.68 to −1.49; *p* = 0.001). The association remained after additional adjustment for baseline RVGLS (β = −3.28 percentage points, 95% CI −5.25 to −1.32; *p* = 0.001). Baseline RVGLS was also associated with subsequent change (β = −0.33 per 1-percentage-point increase in the signed baseline value, 95% CI −0.54 to −0.13; *p* = 0.002). In the sequential model that also included concurrent ΔLVGLS, the ERT coefficient was attenuated but remained statistically significant (β = −2.44 percentage points, 95% CI −4.65 to −0.23; *p* = 0.031; Figure 3C). The association remained significant after adjustment for follow-up duration (*p* = 0.003), restriction to follow-up ≥2 years (*p* < 0.001), and restriction to the IVS4+919G>A subgroup (*p* = 0.003).

## 4. Discussion

In this multicenter Fabry cohort, septal-inclusive RVGLS and free-wall RVFWLS showed different cross-sectional association patterns. RVGLS was associated mainly with LVGLS and IVSd, whereas RVFWLS was associated with RA reservoir strain, and the two RV strain measures were not significantly correlated. Increasing septal hypertrophic burden was accompanied by selective impairment of RVGLS rather than RVFWLS. Longitudinally, ERT-treated patients showed a different RVGLS pattern from untreated patients, while RVFWLS and RA reservoir strain did not show statistically significant between-group changes. These findings support complementary interpretation of the two RV strain measures, but they describe associations and do not establish distinct biological pathways, causal treatment effects, or biological reversibility.

Previous studies established that RV strain may be impaired in Fabry disease, particularly in patients with LV hypertrophy, even when conventional RV systolic indices remain preserved [9,12]. Comparative work also demonstrated that RV strain patterns can differ between Fabry cardiomyopathy and sarcomeric hypertrophic cardiomyopathy [13]. Right-heart and atrial strain abnormalities, including RA remodeling, have likewise been reported [19,20]. The present study also complements reports of left atrial strain changes during disease-specific therapy [21] and the broader emphasis on integrated imaging surveillance in Fabry cardiomyopathy [22,23]. This analysis extends these observations by comparing RVGLS and RVFWLS within the same cohort and with an identical prespecified covariate framework. The selective positive and negative correlations, the different independent correlates, and the longitudinal findings indicate that RVGLS and RVFWLS should not be treated as interchangeable measurements. The contribution of this study is therefore not simply to show that RV strain is abnormal, but to define how the two commonly reported measures relate differently to multichamber remodeling. The segment composition of RVGLS provides a plausible anatomical explanation for its LV-septal associations. RVGLS incorporates septal and free-wall segments, whereas RVFWLS excludes the septum and isolates free-wall deformation [10,24]. Because the interventricular septum is shared by both ventricles and is influenced by LV longitudinal mechanics, hypertrophy, and ventricular interdependence [11], a septal-inclusive measure would be expected to retain information about LV-septal disease burden. This interpretation accords with prior evidence linking LVGLS to myocardial storage and hypertrophy in Fabry disease [25], as well as with the independent associations of RVGLS with LVGLS and IVSd, the graded change in ΔRV strain across IVSd tertiles, and the selective impairment of RVGLS in the LVH subgroup. Earlier multimodality work also related RV involvement in Fabry disease to LV disease burden [12]. These observations provide an anatomically plausible interpretation of the association pattern; they do not prove that septal pathology alone caused the RVGLS findings.

RVFWLS, by excluding the septum, may more closely reflect free-wall mechanics and their interaction with right-sided filling. RA reservoir strain is influenced by venous return, atrial compliance, ventricular systole, and atrioventricular interaction. More generally, regional deformation is shaped by myocardial structure and loading conditions [26,27]. Fabry-related lyso-Gb3 toxicity and inflammatory pathway changes provide a possible disease-level substrate for heterogeneous remodeling, although they were not measured in this cohort [28]. RA functional abnormalities have been described in Fabry disease [20], and RA function has been linked to RV diastolic stiffness in pulmonary arterial hypertension [29]. The association between RVFWLS and RA reservoir strain in the present cohort is therefore consistent with RA–RV interaction. However, it was cross-sectional and does not establish that RA dysfunction caused impaired RVFWLS. Loading conditions, image quality, tracking performance, and other unmeasured features of right-sided remodeling may also have contributed. The longitudinal findings require particularly cautious interpretation. Cardiac damage in Fabry disease evolves across clinically recognizable stages [30]. Disease-specific therapy may stabilize or improve selected cardiac features in Fabry disease, with effects varying by baseline stage, hypertrophic burden, and fibrosis [31,32]. In this cohort, ERT-treated patients had more advanced cardiac involvement at baseline, consistent with treatment selection based on clinically significant organ involvement. This imbalance creates confounding by indication that cannot be eliminated in a nonrandomized study. The association between ERT status and ΔRVGLS persisted after adjustment for baseline RVGLS, follow-up duration, age, sex, and genotype and in analyses restricted by follow-up duration and genotype. These sensitivity analyses support the robustness of the observed association but do not convert it into evidence of a causal treatment effect. The ERT coefficient was attenuated after inclusion of concurrent ΔLVGLS, which is consistent with shared LV-septal mechanical change. Because ΔLVGLS is a concurrent post-baseline change and may lie on the same longitudinal pathway, that sequential model should not be interpreted as conventional baseline confounder adjustment. Systematic reviews similarly describe heterogeneous cardiac findings across disease-specific therapy studies [15,16], while meta-analytic evidence for LVH improvement does not establish parallel recovery across all cardiac compartments [33]. Accordingly, the present result is best described as an ERT status-associated longitudinal pattern. Clinically, these data caution against interpreting change in a single RV strain measure as a global right-ventricular response. RVGLS may partly track shared LV-septal mechanics, whereas RVFWLS may provide complementary information about free-wall and right-sided functional involvement. A multiparametric approach incorporating LVGLS, RVGLS, RVFWLS, and atrial strain may better characterize heterogeneous cardiac remodeling, although its incremental prognostic value remains untested. Until it is validated, this framework should not be used to guide patient management. Prospective studies should combine standardized RV-focused echocardiographic acquisition with CMR native T1, extracellular volume, late gadolinium enhancement, relevant biomarkers, rhythm assessment, and adjudicated clinical outcomes.

This study has several limitations. Its retrospective, nonrandomized design leaves potential residual confounding despite multivariable adjustment and sensitivity analyses. ERT-treated patients had more advanced baseline cardiac involvement, and propensity-based methods would remain limited by the modest sample size, marked treatment-group imbalance in disease severity, and unmeasured determinants of treatment selection. Longitudinal RVFWLS analyses were restricted to studies with adequate paired free-wall tracking, which reduced statistical power and may have introduced selection bias. The cohort predominantly carried the IVS4+919G>A later-onset cardiac variant, limiting generalizability to classical Fabry disease and other GLA variants. Systematic CMR data, including native T1, extracellular volume, and late gadolinium enhancement, were unavailable; biomarkers and clinical outcomes were also not systematically available. Histological and region-specific tissue validation were unavailable. PASP was estimated noninvasively, so the strain/PASP ratios should be considered exploratory load-adjusted indices. The fully automated platform avoided variability from manual contour editing, but measurements remained dependent on image quality, view selection, and tracking performance. Reproducibility estimates reflected repeat image selection and automated-tracing acceptance by the same observer rather than inter-observer or manual-contouring reproducibility.

## 5. Conclusions

RVGLS and RVFWLS showed different association patterns in Fabry cardiomyopathy. Septal-inclusive RVGLS was associated predominantly with LV-septal remodeling, whereas RVFWLS was associated with RA reservoir strain. ERT status was associated with longitudinal RVGLS change, but causal treatment effects and biological reversibility cannot be inferred from this observational study. These hypothesis-generating findings warrant prospective validation using standardized multimodality imaging, relevant biomarkers, and clinically meaningful outcomes.

## Figures and Tables

**Table 1:** Baseline clinical and echocardiographic characteristics.

Variable	All (*n* = 100)	Untreated (*n* = 66)	ERT-Treated (*n* = 34)	*p* Value
**Demographics**				
Age at diagnosis, yr	58.98 ± 15.78	57.53 ± 17.58	61.79 ± 11.23	0.202
Male sex, *n* (%)	64 (64%)	34 (52%)	30 (88%)	0.001 *
Cardiac genotype (IVS4), *n* (%)	91 (91%)	63 (95%)	28 (82%)	0.072
BSA, m^2^	1.73 ± 0.20	1.71 ± 0.21	1.77 ± 0.17	0.199
BMI, kg/m^2^	24.4 ± 4.6	24.6 ± 5.1	24.1 ± 3.6	0.670
**LV Geometry**				
IVSd, mm	11.69 ± 3.72	10.56 ± 3.17	13.87 ± 3.78	<0.001 *
LVPWd, mm	10.88 ± 3.70	9.88 ± 2.75	12.84 ± 4.51	<0.001 *
LV mass, g	251.1 ± 127.5	216.3 ± 113.7	318.7 ± 127.1	<0.001 *
LVIDd, mm	41.72 ± 5.80	41.28 ± 4.76	42.59 ± 7.43	0.294
RWT	0.52 ± 0.18	0.48 ± 0.15	0.59 ± 0.21	0.008 *
**LV Systolic Function**				
LVEF (biplane), %	60.93 ± 10.26	61.64 ± 9.12	59.64 ± 12.11	0.363
LVGLS (3-view), %	−16.18 ± 4.23	−17.12 ± 3.96	−14.37 ± 4.20	0.002 *
**LV Diastolic Function**				
MV E velocity, cm/s	71.44 ± 18.99	73.61 ± 19.66	67.01 ± 17.00	0.114
MV A velocity, cm/s	78.14 ± 22.90	77.80 ± 23.54	78.79 ± 22.00	0.844
E/A ratio	0.96 ± 0.36	0.98 ± 0.33	0.91 ± 0.43	0.362
DecT, ms	204.80 ± 40.52	206.80 ± 39.85	200.45 ± 42.40	0.504
e′ lateral (mitral annulus), cm/s	8.64 ± 3.99	9.12 ± 4.02	7.58 ± 3.78	0.090
e′ septal, cm/s	6.93 ± 2.84	7.52 ± 2.88	5.60 ± 2.27	0.003 *
E/e′ mean	10.03 ± 4.22	9.39 ± 3.74	11.53 ± 4.94	0.033 *
**LA Geometry**				
LAESV (biplane), mL	50.56 ± 27.36	47.74 ± 22.56	55.65 ± 34.32	0.240
**LA Function**				
LA reservoir strain (A4C), %	27.65 ± 12.63	29.00 ± 12.90	25.38 ± 12.00	0.201
**RV Geometry**				
RVIDd basal, mm	30.65 ± 5.01	29.59 ± 5.15	32.70 ± 4.06	0.005 *
RVOT prox, mm	31.60 ± 5.23	31.59 ± 5.31	31.65 ± 5.16	0.958
**RV Function—Septal-Inclusive Measures**				
RVFAC, %	39.92 ± 11.71	40.40 ± 11.48	39.08 ± 12.27	0.632
RVGLS (4C), %	−17.69 ± 5.32	−18.18 ± 5.32	−16.73 ± 5.29	0.199
ΔRV strain (FWLS−GLS), %	−2.40 ± 8.03	−3.23 ± 8.26	−0.99 ± 7.57	0.252
RVGLS/PASP, %·mmHg^−1^	0.70 ± 0.27	0.74 ± 0.25	0.64 ± 0.29	0.195
RVFAC/PASP, %·mmHg^−1^	1.65 ± 0.68	1.68 ± 0.62	1.61 ± 0.81	0.758
**RV Function—Free-Wall Measures**				
RVFWLS, %	−20.22 ± 6.23	−21.32 ± 6.07	−18.35 ± 6.16	0.049 *
RVFWLS/PASP, %·mmHg^−1^	0.78 ± 0.27	0.83 ± 0.27	0.71 ± 0.28	0.174
**Right Atrium**				
RA area, cm^2^	12.64 ± 3.90	12.02 ± 3.54	13.88 ± 4.35	0.032 *
RA strain (reservoir), %	27.98 ± 9.87	28.81 ± 9.94	26.37 ± 9.67	0.242
**Pressure/RV Afterload**				
PASP, mmHg	27.25 ± 7.15	27.02 ± 6.95	27.71 ± 7.67	0.721
TR Vmax, m/s	2.32 ± 0.35	2.30 ± 0.34	2.37 ± 0.38	0.464

Values are mean ± SD or *n* (%). e′ lateral = lateral mitral annular tissue Doppler velocity. ΔRV strain = RVFWLS − RVGLS. RVGLS/PASP, RVFWLS/PASP, and RVFAC/PASP are absolute strain or fractional area change divided by estimated PASP. * *p* < 0.05 between groups.

**Table 2:** Correlations of RV strain measures and related parameters.

Variable Pair	Pearson r	*p* Value
**LV-Septal Associations**		
RVGLS (4C) ↔ LV GLS	+0.735	<0.001 *
RVGLS (4C) ↔ IVSd	+0.580	<0.001 *
RVGLS (4C) ↔ E/e′ mean	+0.431	<0.001 *
RVGLS (4C) ↔ RA strain	+0.075	0.457
RVGLS (4C) ↔ LA reservoir	+0.087	0.428
**Atrial Associations**		
RVFWLS ↔ RA strain	−0.568	<0.001 *
RVFWLS ↔ LA reservoir	−0.452	<0.001 *
RVFWLS ↔ LV GLS	+0.193	0.103
RVFWLS ↔ IVSd	+0.154	0.194
RVFWLS ↔ E/e′ mean	+0.190	0.142
**Between-Measure Association**		
RVGLS (4C) ↔ RVFWLS	+0.053	0.654
**ΔRV Strain Correlates**		
ΔRV ↔ RA strain	−0.496	<0.001 *
ΔRV ↔ LA reservoir	−0.450	<0.001 *
ΔRV ↔ LV GLS	−0.332	0.004 *
ΔRV ↔ IVSd	−0.286	0.014 *
**RV-PA Coupling Indices**		
RVGLS/PASP ↔ LVGLS	−0.664	<0.001 *
RVGLS/PASP ↔ IVSd	−0.549	<0.001 *
RVGLS/PASP ↔ RA strain	−0.051	0.694
RVGLS/PASP ↔ RVFAC	+0.363	0.010 *
RVFWLS/PASP ↔ LVGLS	−0.353	0.022 *
RVFWLS/PASP ↔ IVSd	−0.347	0.025 *
RVFWLS/PASP ↔ RA strain	+0.271	0.082
RVFWLS/PASP ↔ RVFAC	+0.245	0.151

Values are Pearson correlation coefficients. Sample size varies according to data availability. * *p* < 0.05. ΔRV strain, RVFWLS−RVGLS; IVSd, interventricular septal thickness at end-diastole; LA, left atrium; LVGLS, left ventricular global longitudinal strain; PASP, pulmonary artery systolic pressure; RA, right atrium; RVFAC, right ventricular fractional area change; RVFWLS, right ventricular free-wall longitudinal strain; RVGLS, right ventricular global longitudinal strain.

**Table 3:** Multivariable models for RVGLS and RVFWLS.

Predictor	β	95% CI	Std. β	*p* Value	Pattern
**Outcome: RVGLS (4C)** **[R^2^ = 0.536, adj.R^2^ = 0.499]**					
LV GLS	+0.661	[+0.38, +0.94]	+0.466	<0.001 *	LV-septal
IVSd	+0.340	[+0.04, +0.64]	+0.257	0.027 *	LV-septal
E/e′ mean	+0.032	[−0.22, +0.29]	+0.027	0.806	—
RA strain	+0.044	[−0.04, +0.13]	+0.085	0.293	—
Age (years)	+0.035	[−0.03, +0.10]	+0.111	0.297	—
Male sex	+0.165	[−1.71, +2.04]	—	0.861	—
**Outcome: RVFWLS** **[R^2^ = 0.343, adj.R^2^ = 0.270]**					
LV GLS	+0.292	[−0.18, +0.77]	+0.173	0.221	—
IVSd	−0.038	[−0.61, +0.53]	−0.022	0.895	—
E/e′ mean	+0.187	[−0.27, +0.64]	+0.127	0.416	—
RA strain	−0.332	[−0.47, −0.19]	−0.542	<0.001 *	Atrial
Age (years)	−0.058	[−0.17, +0.06]	−0.150	0.316	—
Male sex	+0.830	[−2.43, +4.09]	—	0.612	—

Models included age and sex as clinical covariates in addition to the listed echocardiographic predictors; identical covariates were used for the RVGLS and RVFWLS models. β, unstandardized regression coefficient; CI, confidence interval; Std. β, standardized regression coefficient. * *p* < 0.05.

**Table 4:** Longitudinal Changes in Echocardiographic Parameters According to ERT Status.

Parameter	Untreated (*n* = 35)	ERT-Treated (*n* = 34)	*p* Value	SMD (95% CI)
Septal-Inclusive Measures				
ΔRVGLS (4C), % ^†^	+2.06 ± 3.94	−1.28 ± 3.64	0.017 *	−0.91 [−1.67, −0.15]
ΔLVGLS, % (full cohort)	+2.49 ± 3.71 (*n* = 35)	−0.56 ± 3.04 (*n* = 34)	<0.001 *	−0.90 [−1.39, −0.40]
ΔRVGLS/PASP	−0.054 ± 0.239 (*n* = 19)	+0.049 ± 0.307 (*n* = 17)	0.263	—
Free-Wall and Atrial Measures				
ΔRVFWLS, % ^†^	−0.11 ± 7.37	−0.67 ± 7.97	0.838	−0.07 [−0.76, +0.61]
ΔRA strain, %	−0.25 ± 16.86 (*n* = 35)	−1.14 ± 8.96 (*n* = 34)	0.785	−0.07 [−0.54, +0.41]
Other Parameters				
ΔE/e′ mean	+0.50 ± 2.92 (*n* = 28)	+0.35 ± 2.90 (*n* = 23)	0.856	—
ΔPASP, mmHg	+0.12 ± 6.88 (*n* = 19)	+3.38 ± 10.11 (*n* = 17)	0.262	—

Δ denotes follow-up minus baseline; negative Δ strain values indicate more negative strain at follow-up. CI, confidence interval; ERT, enzyme replacement therapy; PASP, pulmonary artery systolic pressure; RA, right atrial; SMD, standardized mean difference. ^†^ Subset with paired RVGLS and RVFWLS measurements. * *p* < 0.05.

## Data Availability

The data supporting the findings of this study are available from the corresponding authors upon reasonable request. The data are not publicly available because they contain information that could compromise participant privacy.

## Supplementary Materials

The following supporting information can be downloaded at: https://www.mdpi.com/article/10.3390/biom16081196/s1, Table S1: Patient-level GLA variants and clinical classification; Figure S1: Artificial intelligence-based automated right ventricular longitudinal strain analysis.

## References

[1] Tuttolomondo A., Simonetta I., Riolo R., Todaro F., Di Chiara T., Miceli S., Pinto A. (2021). Pathogenesis and Molecular Mechanisms of Anderson-Fabry Disease and Possible New Molecular Addressed Therapeutic Strategies. Int. J. Mol. Sci..

[2] Pieroni M., Moon J.C., Arbustini E., Barriales-Villa R., Camporeale A., Vujkovac A.C., Elliott P.M., Hagege A., Kuusisto J., Linhart A. (2021). Cardiac Involvement in Fabry Disease: JACC Review Topic of the Week. J. Am. Coll. Cardiol..

[3] Pande S., Varzideh F., Gambardella J., Jankauskas S.S., Cerasuolo F.A., Spinelli L., Kansakar U., De Luca A., Kurland I.J., Sidoli S. (2025). Fabry disease cardiomyopathy: A state-of-the-art review. Prog. Cardiovasc. Dis..

[4] Stankowski K., Figliozzi S., Rojanathagoon T., Bampatsias D., Klettas D., Monti L., Bragato R., Masci P.-G., Francone M., Condorelli G. (2025). Imaging predictors of adverse prognosis in Fabry disease cardiomyopathy: A systematic review and meta-analysis. Eur. J. Clin. Investig..

[5] Hsu T.-R., Hung S.-C., Chang F.-P., Yu W.-C., Sung S.-H., Hsu C.-L., Dzhagalov I., Yang C.-F., Chu T.-H., Lee H.-J. (2016). Later Onset Fabry Disease, Cardiac Damage Progress in Silence: Experience with a Highly Prevalent Mutation. J. Am. Coll. Cardiol..

[6] Halfmann M.C., Altmann S., Schoepf U.J., Reichardt C., Hennermann J.B., Kreitner K.-F., Kloeckner R., Hahn F., Dueber C., Varga-Szemes A. (2023). Left atrial strain correlates with severity of cardiac involvement in Anderson-Fabry disease. Eur. Radiol..

[7] Bernardini A., Camporeale A., Pieroni M., Pieruzzi F., Figliozzi S., Lusardi P., Spada M., Mignani R., Burlina A., Carubbi F. (2020). Atrial Dysfunction Assessed by Cardiac Magnetic Resonance as an Early Marker of Fabry Cardiomyopathy. JACC Cardiovasc. Imaging.

[8] Pucci M., Iadevaia V., Gammaldi V., Iervolino A., Capece L.M., Sciascia D., Cuomo V., Iacono M., Paoletta D., Santoro C. (2023). Right Ventricular Myocardial Involvement in Anderson-Fabry Disease at Diagnosis: Evaluation with Three-Dimensional Strain Imaging. Life.

[9] Lillo R., Graziani F., Panaioli E., Mencarelli E., Pieroni M., Camporeale A., Manna R., Sicignano L.L., Verrecchia E., Lombardo A. (2021). Right ventricular strain in Anderson-Fabry disease. Int. J. Cardiol..

[10] Badano L.P., Kolias T.J., Muraru D., Abraham T.P., Aurigemma G., Edvardsen T., D’Hooge J., Donal E., Fraser A.G., Marwick T. (2018). Standardization of left atrial, right ventricular, and right atrial deformation imaging using two-dimensional speckle tracking echocardiography: A consensus document of the EACVI/ASE/Industry Task Force to standardize deformation imaging. Eur. Heart J. Cardiovasc. Imaging.

[11] Sanz J., Sánchez-Quintana D., Bossone E., Bogaard H.J., Naeije R. (2019). Anatomy, Function, and Dysfunction of the Right Ventricle: JACC State-of-the-Art Review. J. Am. Coll. Cardiol..

[12] Niemann M., Breunig F., Beer M., Herrmann S., Strotmann J., Hu K., Emmert A., Voelker W., Ertl G., Wanner C. (2010). The right ventricle in Fabry disease: Natural history and impact of enzyme replacement therapy. Heart.

[13] Meucci M.C., Lillo R., Lombardo A., Lanza G.A., Bootsma M., Butcher S.C., Massetti M., Manna R., Bax J.J., Crea F. (2023). Comparative analysis of right ventricular strain in Fabry cardiomyopathy and sarcomeric hypertrophic cardiomyopathy. Eur. Heart J. Cardiovasc. Imaging.

[14] Voigt J.-U., Cvijic M. (2019). 2- and 3-Dimensional Myocardial Strain in Cardiac Health and Disease. JACC Cardiovasc. Imaging.

[15] Orsborne C., Black N., Naish J.H., Woolfson P., Reid A.B., Schmitt M., Jovanovic A., Miller C.A. (2023). Disease-specific therapy for the treatment of the cardiovascular manifestations of Fabry disease: A systematic review. Heart.

[16] Jovanovic A., Miller-Hodges E., Castriota F., Evuarherhe O., Ayodele O., Hughes D., Pintos-Morell G., Giugliani R., Feriozzi S., Siffel C. (2025). Clinical Efficacy and Real-World Effectiveness of Fabry Disease Treatments: A Systematic Literature Review. J. Clin. Med..

[17] Myhre P.L., Hung C.-L., Frost M.J., Jiang Z., Ouwerkerk W., Teramoto K., Svedlund S., Saraste A., Hage C., Tan R.-S. (2024). External validation of a deep learning algorithm for automated echocardiographic strain measurements. Eur. Heart J. Digit. Health.

[18] Lang R.M., Badano L.P., Mor-Avi V., Afilalo J., Armstrong A., Ernande L., Flachskampf F.A., Foster E., Goldstein S.A., Kuznetsova T. (2015). Recommendations for cardiac chamber quantification by echocardiography in adults: An update from the American Society of Echocardiography and the European Association of Cardiovascular Imaging. Eur. Heart J. Cardiovasc. Imaging.

[19] Mattig I., Steudel T., Klingel K., Barzen G., Frumkin D., Spethmann S., Romero Dorta E., Stangl K., Heidecker B., Landmesser U. (2024). Right heart and left atrial strain to differentiate cardiac amyloidosis and Fabry disease. Sci. Rep..

[20] Lillo R., Cianci A., Meucci M.C., Iannaccone G., Di Brango C., Tusa F., Marsilia M., Lanza G.A., Lombardo A., Burzotta F. (2025). Right atrial strain in Anderson-Fabry disease. Front. Cardiovasc. Med..

[21] Pogoda C., Brand S.-M., Duning T., Schmidt-Pogoda A., Sindermann J., Lenders M., Brand E. (2023). Impact of enzyme replacement therapy and migalastat on left atrial strain and cardiomyopathy in patients with Fabry disease. Front. Cardiovasc. Med..

[22] Perry R., Shah R., Saiedi M., Patil S., Ganesan A., Linhart A., Selvanayagam J.B. (2019). The Role of Cardiac Imaging in the Diagnosis and Management of Anderson-Fabry Disease. JACC Cardiovasc. Imaging.

[23] Linhart A., Germain D.P., Olivotto I., Akhtar M.M., Anastasakis A., Hughes D., Namdar M., Pieroni M., Hagège A., Cecchi F. (2020). An expert consensus document on the management of cardiovascular manifestations of Fabry disease. Eur. J. Heart Fail..

[24] Mukherjee M., Rudski L.G., Addetia K., Afilalo J., D’Alto M., Freed B.H., Friend L.B., Gargani L., Grapsa J., Hassoun P.M. (2025). Guidelines for the Echocardiographic Assessment of the Right Heart in Adults and Special Considerations in Pulmonary Hypertension: Recommendations from the American Society of Echocardiography. J. Am. Soc. Echocardiogr..

[25] Vijapurapu R., Nordin S., Baig S., Liu B., Rosmini S., Augusto J., Tchan M., Hughes D.A., Geberhiwot T., Moon J.C. (2019). Global longitudinal strain, myocardial storage and hypertrophy in Fabry disease. Heart.

[26] Pagourelias E.D., Mirea O., Vovas G., Duchenne J., Michalski B., Van Cleemput J., Bogaert J., Vassilikos V.P., Voigt J.-U. (2019). Relation of regional myocardial structure and function in hypertrophic cardiomyopathy and amyloidois: A combined two-dimensional speckle tracking and cardiovascular magnetic resonance analysis. Eur. Heart J. Cardiovasc. Imaging.

[27] Mihos C.G., Liu J.E., Anderson K.M., Pernetz M.A., O’Driscoll J.M., Aurigemma G.P., Ujueta F., Wessly P., on behalf of American Heart Association Council on Peripheral Vascular Disease, Council on Cardiovascular and Stroke Nursing, and Council on Clinical Cardiology (2025). Speckle-Tracking Strain Echocardiography for the Assessment of Left Ventricular Structure and Function: A Scientific Statement from the American Heart Association. Circulation.

[28] Nikolaenko V., Warnock D.G., Mills K., Heywood W.E. (2023). Elucidating the toxic effect and disease mechanisms associated with Lyso-Gb3 in Fabry disease. Hum. Mol. Genet..

[29] Wessels J.N., Mouratoglou S.A., van Wezenbeek J., Handoko M.L., Marcus J.T., Meijboom L.J., Westerhof B.E., Bogaard H.J., Strijkers G.J., Vonk Noordegraaf A. (2022). Right atrial function is associated with right venticular diastolic stiffness: RA-RV interaction in pulmonary arterial hypertension. Eur. Respir. J..

[30] Lillo R., Meucci M.C., Del Franco A., Monda E., Iannaccone G., Ditaranto R., Di Nicola F., Serratore S., Lombardo A., Spinelli L. (2025). Evolution of Cardiac Damage Staged with Echocardiography in Fabry Disease. J. Am. Heart Assoc..

[31] Frustaci A., Verardo R., Galea N., Alfarano M., Magnocavallo M., Marchitelli L., Sansone L., Belli M., Cristina M., Frustaci E. (2024). Long-Term Clinical-Pathologic Results of Enzyme Replacement Therapy in Prehypertrophic Fabry Disease Cardiomyopathy. J. Am. Heart Assoc..

[32] Weidemann F., Niemann M., Breunig F., Herrmann S., Beer M., Störk S., Voelker W., Ertl G., Wanner C., Strotmann J. (2009). Long-term effects of enzyme replacement therapy on fabry cardiomyopathy: Evidence for a better outcome with early treatment. Circulation.

[33] Lee C.-L., Lin S.-P., Niu D.-M., Lin H.-Y. (2022). Fabry Disease and the Effectiveness of Enzyme Replacement Therapy (ERT) in Left Ventricular Hypertrophy (LVH) Improvement: A Review and Meta-Analysis. Int. J. Med. Sci..

