# Supplementary material for: Two Faces of the Right Ventricle in Fabry Cardiomyopathy: Septal-Coupled and Atrial-Coupled Strain Patterns and Their Differential Associations with Enzyme Replacement Therapy
Source: Biomolecules. 2026 Aug 17;16(8):1196. doi: 10.3390/biom16081196 (PMC13510769; doi:10.3390/biom16081196)
Supplement: Supplementary file 1 [file biomolecules-16-01196-s001.zip › biomolecules-4398463-supplementary.pdf]

Supplementary Table S1. Patient-level GLA variants and clinical classification

Variants are reported using transcript NM_000169.3. Study IDs are de-identified. Clinical classifications reflect the aggregate germline interpretation in ClinVar. ERT, enzyme replacement therapy.

Study ID	Sex	GLA variant (HGVS)	Protein consequence / alias	ClinVar classification	Phenotype	Treatment status
1	Female	NM_000169.3(GLA):c.640-801G>A	Intronic (IVS4+919G>A)	Pathogenic	Later-onset cardiac	ERT-treated
2	Male	NM_000169.3(GLA):c.640-801G>A	Intronic (IVS4+919G>A)	Pathogenic	Later-onset cardiac	Untreated
3	Male	NM_000169.3(GLA):c.640-801G>A	Intronic (IVS4+919G>A)	Pathogenic	Later-onset cardiac	Untreated
4	Female	NM_000169.3(GLA):c.640-801G>A	Intronic (IVS4+919G>A)	Pathogenic	Later-onset cardiac	Untreated
5	Female	NM_000169.3(GLA):c.640-801G>A	Intronic (IVS4+919G>A)	Pathogenic	Later-onset cardiac	Untreated
6	Female	NM_000169.3(GLA):c.640-801G>A	Intronic (IVS4+919G>A)	Pathogenic	Later-onset cardiac	Untreated
7	Female	NM_000169.3(GLA):c.640-801G>A	Intronic (IVS4+919G>A)	Pathogenic	Later-onset cardiac	ERT-treated
8	Male	NM 000169.3(GLA):c.640-801G>A	Intronic (IVS4+919G>A)	Pathogenic	Later-onset cardiac	Untreated
9	Female	NM_000169.3(GLA):c.640-801G>A	Intronic (IVS4+919G>A)	Pathogenic	Later-onset cardiac	Untreated
10	Female	NM_000169.3(GLA):c.640-801G>A	Intronic (IVS4+919G>A)	Pathogenic	Later-onset cardiac	Untreated
11	Female	NM_000169.3(GLA):c.640-801G>A	Intronic (IVS4+919G>A)	Pathogenic	Later-onset cardiac	Untreated
12	Female	NM_000169.3(GLA):c.640-801G>A	Intronic (IVS4+919G>A)	Pathogenic	Later-onset cardiac	Untreated
13	Female	NM_000169.3(GLA):c.640-801G>A	Intronic (IVS4+919G>A)	Pathogenic	Later-onset cardiac	Untreated
14	Female	NM_000169.3(GLA):c.640-801G>A	Intronic (IVS4+919G>A)	Pathogenic	Later-onset cardiac	Untreated
15	Male	NM_000169.3(GLA):c.640-801G>A	Intronic (IVS4+919G>A)	Pathogenic	Later-onset cardiac	Untreated
16	Female	NM_000169.3(GLA):c.640-801G>A	Intronic (IVS4+919G>A)	Pathogenic	Later-onset cardiac	Untreated
17	Male	NM_000169.3(GLA):c.640-801G>A	Intronic (IVS4+919G>A)	Pathogenic	Later-onset cardiac	ERT-treated
18	Male	NM_000169.3(GLA):c.640-801G>A	Intronic (IVS4+919G>A)	Pathogenic	Later-onset cardiac	ERT-treated
19	Female	NM_000169.3(GLA):c.640-801G>A	Intronic (IVS4+919G>A)	Pathogenic	Later-onset cardiac	Untreated
20	Female	NM_000169.3(GLA):c.640-801G>A	Intronic (IVS4+919G>A)	Pathogenic	Later-onset cardiac	Untreated
21	Male	NM 000169.3(GLA):c.640-801G>A	Intronic (IVS4+919G>A)	Pathogenic	Later-onset cardiac	ERT-treated
22	Female	NM_000169.3(GLA):c.640-801G>A	Intronic (IVS4+919G>A)	Pathogenic	Later-onset cardiac	Untreated
23	Male	NM_000169.3(GLA):c.640-801G>A	Intronic (IVS4+919G>A)	Pathogenic	Later-onset cardiac	ERT-treated
24	Female	NM_000169.3(GLA):c.640-801G>A	Intronic (IVS4+919G>A)	Pathogenic	Later-onset cardiac	Untreated
25	Male	NM_000169.3(GLA):c.640-801G>A	Intronic (IVS4+919G>A)	Pathogenic	Later-onset cardiac	Untreated
26	Female	NM_000169.3(GLA):c.640-801G>A	Intronic (IVS4+919G>A)	Pathogenic	Later-onset cardiac	Untreated
27	Female	NM_000169.3(GLA):c.640-801G>A	Intronic (IVS4+919G>A)	Pathogenic	Later-onset cardiac	Untreated
28	Female	NM_000169.3(GLA):c.640-801G>A	Intronic (IVS4+919G>A)	Pathogenic	Later-onset cardiac	Untreated
29	Female	NM_000169.3(GLA):c.640-801G>A	Intronic (IVS4+919G>A)	Pathogenic	Later-onset cardiac	Untreated
30	Male	NM_000169.3(GLA):c.640-801G>A	Intronic (IVS4+919G>A)	Pathogenic	Later-onset cardiac	Untreated
31	Female	NM_000169.3(GLA):c.640-801G>A	Intronic (IVS4+919G>A)	Pathogenic	Later-onset cardiac	Untreated

Study ID	Sex	GLA variant (HGVS)	Protein consequence / alias	ClinVar classification	Phenotype	Treatment status
32	Male	NM_000169.3(GLA):c.640-801G>A	Intronic (IVS4+919G>A)	Pathogenic	Later-onset cardiac	Untreated
33	Male	NM_000169.3(GLA):c.640-801G>A	Intronic (IVS4+919G>A)	Pathogenic	Later-onset cardiac	Untreated
34	Male	NM_000169.3(GLA):c.640-801G>A	Intronic (IVS4+919G>A)	Pathogenic	Later-onset cardiac	Untreated
35	Male	NM_000169.3(GLA):c.640-801G>A	Intronic (IVS4+919G>A)	Pathogenic	Later-onset cardiac	ERT-treated
36	Male	NM_000169.3(GLA):c.1228A>G	p.Thr410Ala	Pathogenic	Classical	Untreated
37	Female	NM_000169.3(GLA):c.640-801G>A	Intronic (IVS4+919G>A)	Pathogenic	Later-onset cardiac	ERT-treated
38	Male	NM_000169.3(GLA):c.1066C>T	p.Arg356Trp	Pathogenic/Likely pathogenic	Classical	Untreated
39	Female	NM_000169.3(GLA):c.640-801G>A	Intronic (IVS4+919G>A)	Pathogenic	Later-onset cardiac	Untreated
40	Female	NM_000169.3(GLA):c.640-801G>A	Intronic (IVS4+919G>A)	Pathogenic	Later-onset cardiac	Untreated
41	Female	NM_000169.3(GLA):c.640-801G>A	Intronic (IVS4+919G>A)	Pathogenic	Later-onset cardiac	ERT-treated
42	Male	NM_000169.3(GLA):c.640-801G>A	Intronic (IVS4+919G>A)	Pathogenic	Later-onset cardiac	ERT-treated
43	Male	NM_000169.3(GLA):c.640-801G>A	Intronic (IVS4+919G>A)	Pathogenic	Later-onset cardiac	ERT-treated
44	Male	NM_000169.3(GLA):c.640-801G>A	Intronic (IVS4+919G>A)	Pathogenic	Later-onset cardiac	Untreated
45	Female	NM_000169.3(GLA):c.640-801G>A	Intronic (IVS4+919G>A)	Pathogenic	Later-onset cardiac	Untreated
46	Female	NM_000169.3(GLA):c.640-801G>A	Intronic (IVS4+919G>A)	Pathogenic	Later-onset cardiac	Untreated
47	Male	NM_000169.3(GLA):c.640-801G>A	Intronic (IVS4+919G>A)	Pathogenic	Later-onset cardiac	ERT-treated
48	Female	NM_000169.3(GLA):c.640-801G>A	Intronic (IVS4+919G>A)	Pathogenic	Later-onset cardiac	Untreated
49	Male	NM_000169.3(GLA):c.640-801G>A	Intronic (IVS4+919G>A)	Pathogenic	Later-onset cardiac	ERT-treated
50	Male	NM_000169.3(GLA):c.640-801G>A	Intronic (IVS4+919G>A)	Pathogenic	Later-onset cardiac	ERT-treated
51	Male	NM_000169.3(GLA):c.640-801G>A	Intronic (IVS4+919G>A)	Pathogenic	Later-onset cardiac	ERT-treated
52	Male	NM_000169.3(GLA):c.640-801G>A	Intronic (IVS4+919G>A)	Pathogenic	Later-onset cardiac	Untreated
53	Male	NM_000169.3(GLA):c.640-801G>A	Intronic (IVS4+919G>A)	Pathogenic	Later-onset cardiac	Untreated
54	Male	NM_000169.3(GLA):c.640-801G>A	Intronic (IVS4+919G>A)	Pathogenic	Later-onset cardiac	ERT-treated
55	Male	NM_000169.3(GLA):c.640-801G>A	Intronic (IVS4+919G>A)	Pathogenic	Later-onset cardiac	ERT-treated
56	Female	NM_000169.3(GLA):c.640-801G>A	Intronic (IVS4+919G>A)	Pathogenic	Later-onset cardiac	Untreated
57	Female	NM_000169.3(GLA):c.640-801G>A	Intronic (IVS4+919G>A)	Pathogenic	Later-onset cardiac	Untreated
58	Female	NM_000169.3(GLA):c.640-801G>A	Intronic (IVS4+919G>A)	Pathogenic	Later-onset cardiac	Untreated
59	Male	NM_000169.3(GLA):c.640-801G>A	Intronic (IVS4+919G>A)	Pathogenic	Later-onset cardiac	Untreated
60	Male	NM_000169.3(GLA):c.640-801G>A	Intronic (IVS4+919G>A)	Pathogenic	Later-onset cardiac	Untreated
61	Male	NM_000169.3(GLA):c.640-801G>A	Intronic (IVS4+919G>A)	Pathogenic	Later-onset cardiac	Untreated
62	Female	NM_000169.3(GLA):c.640-801G>A	Intronic (IVS4+919G>A)	Pathogenic	Later-onset cardiac	Untreated
63	Female	NM_000169.3(GLA):c.640-801G>A	Intronic (IVS4+919G>A)	Pathogenic	Later-onset cardiac	Untreated
64	Male	NM_000169.3(GLA):c.640-801G>A	Intronic (IVS4+919G>A)	Pathogenic	Later-onset cardiac	ERT-treated
65	Male	NM_000169.3(GLA):c.640-801G>A	Intronic (IVS4+919G>A)	Pathogenic	Later-onset cardiac	Untreated
66	Male	NM_000169.3(GLA):c.640-801G>A	Intronic (IVS4+919G>A)	Pathogenic	Later-onset cardiac	ERT-treated

Study ID	Sex	GLA variant (HGVS)	Protein consequence / alias	ClinVar classification	Phenotype	Treatment status
67	Male	NM_000169.3(GLA):c.413G>A	p.Gly138Glu	Pathogenic	Classical	ERT-treated
68	Male	NM_000169.3(GLA):c.640-801G>A	Intronic (IVS4+919G>A)	Pathogenic	Later-onset cardiac	Untreated
69	Male	NM_000169.3(GLA):c.640-801G>A	Intronic (IVS4+919G>A)	Pathogenic	Later-onset cardiac	Untreated
70	Male	NM_000169.3(GLA):c.640-801G>A	Intronic (IVS4+919G>A)	Pathogenic	Later-onset cardiac	Untreated
71	Male	NM_000169.3(GLA):c.640-801G>A	Intronic (IVS4+919G>A)	Pathogenic	Later-onset cardiac	Untreated
72	Female	NM_000169.3(GLA):c.640-801G>A	Intronic (IVS4+919G>A)	Pathogenic	Later-onset cardiac	Untreated
73	Male	NM_000169.3(GLA):c.640-801G>A	Intronic (IVS4+919G>A)	Pathogenic	Later-onset cardiac	Untreated
74	Male	NM_000169.3(GLA):c.1228A>G	p.Thr410Ala	Pathogenic	Classical	ERT-treated
75	Male	NM_000169.3(GLA):c.640-801G>A	Intronic (IVS4+919G>A)	Pathogenic	Later-onset cardiac	ERT-treated
76	Male	NM_000169.3(GLA):c.640-801G>A	Intronic (IVS4+919G>A)	Pathogenic	Later-onset cardiac	Untreated
77	Male	NM_000169.3(GLA):c.640-801G>A	Intronic (IVS4+919G>A)	Pathogenic	Later-onset cardiac	Untreated
78	Male	NM_000169.3(GLA):c.640-801G>A	Intronic (IVS4+919G>A)	Pathogenic	Later-onset cardiac	ERT-treated
79	Male	NM_000169.3(GLA):c.640-801G>A	Intronic (IVS4+919G>A)	Pathogenic	Later-onset cardiac	Untreated
80	Male	NM_000169.3(GLA):c.640-801G>A	Intronic (IVS4+919G>A)	Pathogenic	Later-onset cardiac	ERT-treated
81	Male	NM_000169.3(GLA):c.640-801G>A	Intronic (IVS4+919G>A)	Pathogenic	Later-onset cardiac	Untreated
82	Male	NM_000169.3(GLA):c.1066C>T	p.Arg356Trp	Pathogenic/Likely pathogenic	Classical	ERT-treated
83	Male	NM_000169.3(GLA):c.640-801G>A	Intronic (IVS4+919G>A)	Pathogenic	Later-onset cardiac	ERT-treated
84	Male	NM_000169.3(GLA):c.640-801G>A	Intronic (IVS4+919G>A)	Pathogenic	Later-onset cardiac	ERT-treated
85	Female	NM_000169.3(GLA):c.640-801G>A	Intronic (IVS4+919G>A)	Pathogenic	Later-onset cardiac	Untreated
86	Female	NM_000169.3(GLA):c.640-801G>A	Intronic (IVS4+919G>A)	Pathogenic	Later-onset cardiac	Untreated
87	Male	NM_000169.3(GLA):c.1066C>T	p.Arg356Trp	Pathogenic/Likely pathogenic	Classical	Untreated
88	Male	NM_000169.3(GLA):c.640-801G>A	Intronic (IVS4+919G>A)	Pathogenic	Later-onset cardiac	Untreated
89	Male	NM_000169.3(GLA):c.640-801G>A	Intronic (IVS4+919G>A)	Pathogenic	Later-onset cardiac	Untreated
90	Male	NM_000169.3(GLA):c.640-801G>A	Intronic (IVS4+919G>A)	Pathogenic	Later-onset cardiac	Untreated
91	Male	NM_000169.3(GLA):c.640-801G>A	Intronic (IVS4+919G>A)	Pathogenic	Later-onset cardiac	ERT-treated
92	Male	NM_000169.3(GLA):c.658C>T	p.Arg220Ter	Pathogenic	Classical	ERT-treated
93	Male	NM_000169.3(GLA):c.640-801G>A	Intronic (IVS4+919G>A)	Pathogenic	Later-onset cardiac	ERT-treated
94	Male	NM_000169.3(GLA):c.640-801G>A	Intronic (IVS4+919G>A)	Pathogenic	Later-onset cardiac	Untreated
95	Male	NM_000169.3(GLA):c.640-801G>A	Intronic (IVS4+919G>A)	Pathogenic	Later-onset cardiac	Untreated
96	Male	NM_000169.3(GLA):c.640-801G>A	Intronic (IVS4+919G>A)	Pathogenic	Later-onset cardiac	Untreated
97	Male	NM_000169.3(GLA):c.658C>T	p.Arg220Ter	Pathogenic	Classical	ERT-treated
98	Male	NM_000169.3(GLA):c.658C>T	p.Arg220Ter	Pathogenic	Classical	ERT-treated
99	Male	NM_000169.3(GLA):c.640-801G>A	Intronic (IVS4+919G>A)	Pathogenic	Later-onset cardiac	ERT-treated
100	Male	NM_000169.3(GLA):c.640-801G>A	Intronic (IVS4+919G>A)	Pathogenic	Later-onset cardiac	ERT-treated

Supplementary Figure S1. Artificial intelligence–based automated right ventricular longitudinal strain analysis

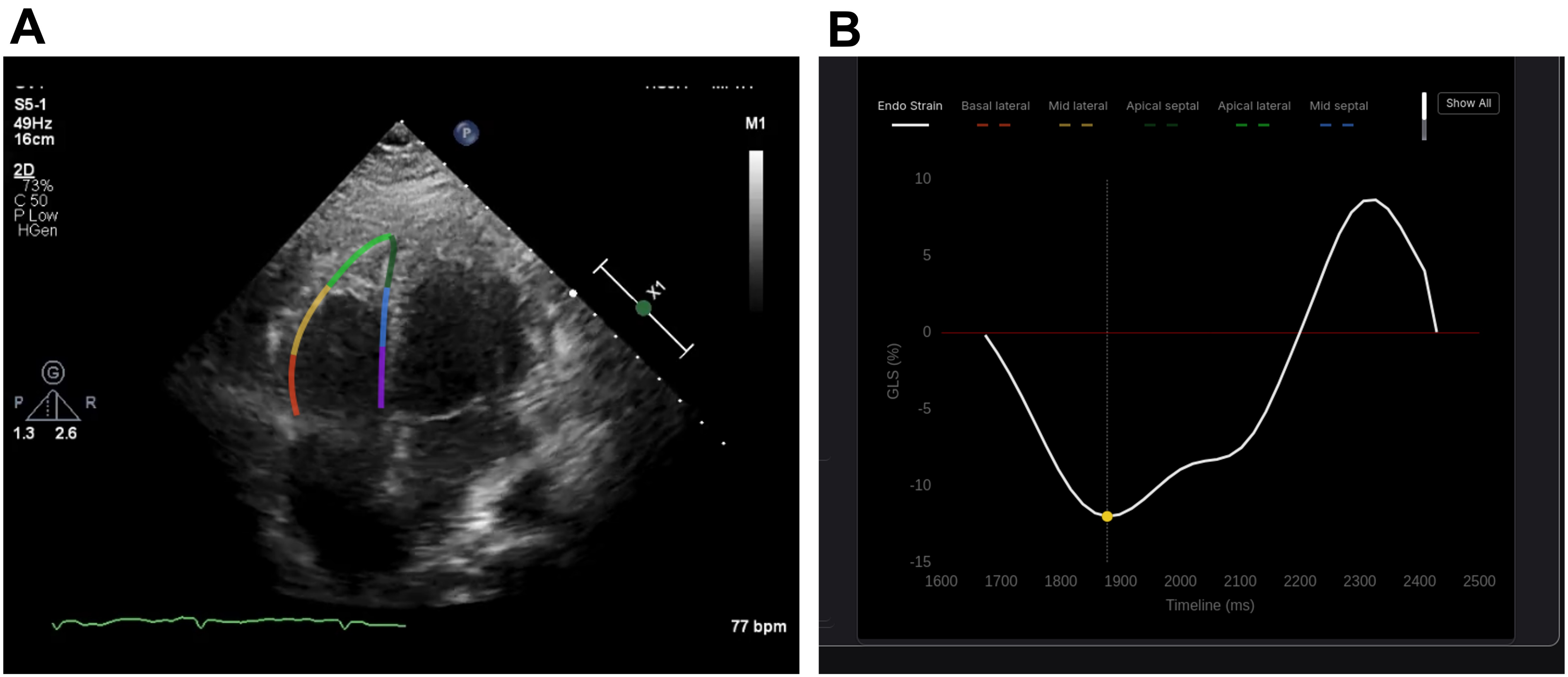

(A) Right ventricle–focused apical four-chamber view showing the automatically generated, color-coded endocardial tracking contours of the right ventricular free wall and interventricular septum. (B) Corresponding longitudinal strain curve automatically generated throughout the cardiac cycle. The peak negative strain is indicated by the yellow marker. RVGLS was derived from both the septal and free-wall segments, whereas RVFWLS was derived from the free-wall segments only. All contours and strain curves were generated automatically using Us2.ai without manual contour correction. AI, artificial intelligence; RV, right ventricle; GLS, global longitudinal strain; RVGLS, right ventricular global longitudinal strain; RVFWLS, right ventricular free-wall longitudinal strain.
